# Trial Design for Cancer Immunotherapy: A Methodological Toolkit

**DOI:** 10.3390/cancers15184669

**Published:** 2023-09-21

**Authors:** Everardo D. Saad, Elisabeth Coart, Vaiva Deltuvaite-Thomas, Leandro Garcia-Barrado, Tomasz Burzykowski, Marc Buyse

**Affiliations:** 1 International Drug Development Institute, Louvain-la-Neuve (IDDI), 1340 Ottignies-Louvain-la-Neuve, Belgium; elisabeth.coart@iddi.com (E.C.); vaiva.thomas@iddi.com (V.D.-T.); leandro.garciabarrado@iddi.com (L.G.-B.); tomasz.burzykowski@iddi.com (T.B.); marc.buyse@iddi.com (M.B.); 2 Interuniversity Institute for Biostatistics and Statistical Bioinformatics (I-BioStat), Hasselt University, B-3500 Hasselt, Belgium

**Keywords:** immunotherapy, checkpoint inhibitors, trial design

## Abstract

**Simple Summary:**

Immunotherapy has become a very important treatment against several types of cancer. The clinical trials of immunotherapy have shown that these treatments present us with some novel challenges in terms of the methodology required to assess their efficacy and safety. In this article, we discuss what we consider to be the most important among those challenges and provide some suggestions to address them in the design and analysis of clinical trials of immunotherapy, especially with the class of treatments named “checkpoint inhibitors”. In summary, the methodological aspects discussed in this article refer to definitions and implementation of efficacy endpoints, the evaluation of safety, and specific statistical issues that may require special attention in these trials.

**Abstract:**

Immunotherapy with checkpoint inhibitors (CPIs) and cell-based products has revolutionized the treatment of various solid tumors and hematologic malignancies. These agents have shown unprecedented response rates and long-term benefits in various settings. These clinical advances have also pointed to the need for new or adapted approaches to trial design and assessment of efficacy and safety, both in the early and late phases of drug development. Some of the conventional statistical methods and endpoints used in other areas of oncology appear to be less appropriate in immuno-oncology. Conversely, other methods and endpoints have emerged as alternatives. In this article, we discuss issues related to trial design in the early and late phases of drug development in immuno-oncology, with a focus on CPIs. For early trials, we review the most salient issues related to dose escalation, use and limitations of tumor response and progression criteria for immunotherapy, the role of duration of response as an endpoint in and of itself, and the need to conduct randomized trials as early as possible in the development of new therapies. For late phases, we discuss the choice of primary endpoints for randomized trials, review the current status of surrogate endpoints, and discuss specific statistical issues related to immunotherapy, including non-proportional hazards in the assessment of time-to-event endpoints, alternatives to the Cox model in these settings, and the method of generalized pairwise comparisons, which can provide a patient-centric assessment of clinical benefit and be used to design randomized trials.

## 1. Introduction

Immunotherapy with ocheckpoint inhibitors (CPIs) has led to unprecedented responses in tumor types marked by resistance to conventional treatments as well as to improvements in long-term outcomes in phase 3 trials from the use [1,2,3]. However, several issues related to the assessment of efficacy and safety have emerged that highlight differences between immunotherapy and other treatment modalities and that have an impact on the design, analysis, and interpretation of early-phase and late-phase trials for the development of novel immunotherapy agents; such issues have been discussed in previous reviews [4,5,6,7,8,9,10,11,12,13]. Foremost among these issues are the atypical dynamics of tumor response and disease progression, the often-atypical behavior of Kaplan–Meier curves for progression-free (PFS) and overall survival (OS), and the kinetics of immune-related adverse events (IRAEs), but other issues have also been identified. Given the increasing proportion of oncology trials dedicated to immunotherapy, it is important to consider these issues and the methods that have been proposed for addressing them. In this article, we provide an overview of the most salient methodological issues with the aim of providing clinicians, clinical trialists, statisticians, and sponsors with actionable tools that may help design immunotherapy trials, with a greater emphasis on CPIs. We divide the article into two large parts: the first dedicated to fundamental knowledge pertaining to differences between immunotherapy and other modalities, and the second to specific decisions and choices related to trial design in early and late phases. Issues that affect both of these phases will only be discussed where we find a given issue to be more pressing. Unless otherwise specified, early-phase trials denote phase 1 trials with or without expansion cohorts, as well as phase 2 trials with no formal comparative intent, whereas randomized phase 2 trials with a comparative intent and phase 3 trials are considered late-phase trials. Our focus is advanced disease, as this is the typical setting for developing novel agents in oncology.

## 2. Fundamental Considerations

### 2.1. Mechanistic Aspects of Immunotherapy

An antitumor effect that can be directly measured is usually considered a sine qua non of effective therapy in oncology. As with other modalities, tumor responses are a desirable first step in the development of novel immunotherapy agents, as well as a valid indicator of treatment benefit in individual patients. In addition, the objective response rate (ORR) is useful for the efficacy assessment of single-arm trials and for comparing different treatments in a randomized setting [14]. However, immunotherapy works indirectly, and its effects comprise a continuum of biological interactions between the immune system and tumor cells [1,15]. Importantly, tumor infiltration by cytotoxic T lymphocytes and other effector immune cells is required for the antitcancer activity of immunotherapy [16,17,18]. Such activity is usually balanced by immune-suppression mechanisms that act in the tumor microenvironment [15,16]. The concept of immunoediting can be used to describe the interactions between the immune system and the tumor, as well as the dynamic and variable nature of such interactions over time [17]. According to this concept, there are three states that characterize the end result of interactions between the immune system and the tumor: elimination, equilibrium, and escape. An interesting parallel has been suggested between these three states and the clinical phenomena of response, disease stability, and disease progression after immunotherapy [1]. Therefore, the mechanisms underlying response and resistance to immunotherapy may help explain the observed kinetics of tumor shrinkage and growth during treatment. Such dynamics probably underlie the somewhat specific behavior of PFS and OS with immunotherapy. Likewise, the profile and the kinetics of IRAEs are also thought to be related to the unique mechanism of action of immunotherapy [19].

### 2.2. Response Assessment with Immunotherapy

#### 2.2.1. Unique Patterns of Response and Progression

**Figure 1:**
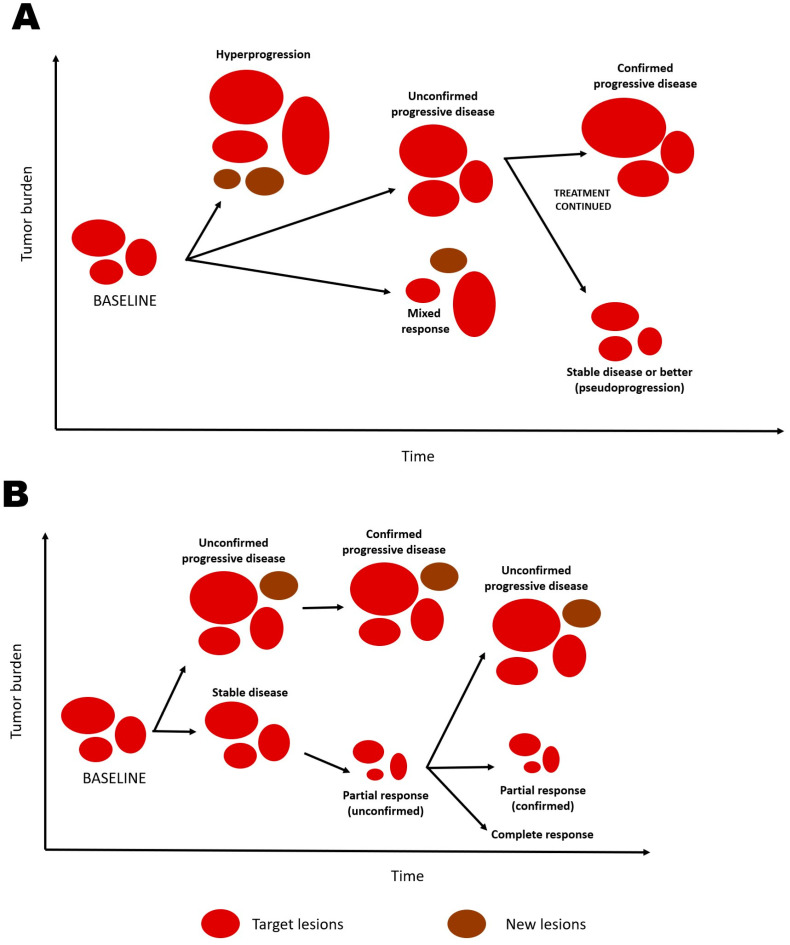
Schematic representation of selected unusual (**A**) and usual (**B**) response patterns in immunotherapy.

Experience to date suggests that responses in most patients treated with immunotherapy behave qualitatively as with other treatment modalities. Quantitatively, the ORR of different CPIs varies between less than 10% and slightly over 60% according to cancer type and line [20] and is on average close to 30% for agents approved by the Food and Drug Administration (FDA) [21]. Nevertheless, unusual patterns of response may be observed with immunotherapy, and the mechanistic considerations made above may underlie some of these patterns. Three unusual patterns of response have been described: mixed responses, pseudoprogression, and hyperprogression [1,12]. Figure 1 depicts these phenomena schematically. The heterogeneity of tumors and their immunological landscape probably underlies cases of mixed responses, in which some lesions shrink and others remain stable or grow [1,12,22]. The frequency and prognostic meaning of mixed responses require further investigation. On the other hand, an initial tumor growth is followed by bona fide responses in 2% to 9% of patients treated with a CPI, if treatment is continued beyond progressive disease (PD); this phenomenon has been termed pseudoprogression [12,23,24]. In some of these cases, the initial increase in the volume of a lesion destined to shrink is probably due to lymphocytic infiltration of tumors, but a delayed action of immunotherapy has also been postulated as a potential explanation for the phenomenon [12,23,25]. The fact that pseudoprogression is associated with favorable outcomes, when compared with PD, lends further support for these postulated mechanisms, at least in advanced melanoma treated with CPIs [23,26,27]. Therefore, the response profile among patients treated with CPIs may not be adequately captured by the Response Evaluation Criteria in Solid Tumors (RECIST 1.1 [28]) [1,12,24]. Another unusual phenomenon is hyperprogression, characterized by very early signs of unquestionable PD after treatment in some patients [12,29,30]. The frequency of hyperprogression is not yet defined but is likely in the vicinity of 7–9% [29,30]. Although definitions may vary, hyperprogression has been associated with unfavorable outcomes [12,29,31,32]. While hyperprogression has been postulated to underlie the early detriment of the use of CPIs [12], a putative immunological mechanism for hyperprogression remains to be found.

#### 2.2.2. Response Criteria for Immunotherapy

The unusual response patterns just discussed raised concerns about the adequacy of RECIST early in the development of CPIs [23,25]. Such concerns led to the publication of the immune-related response criteria (irRC), which were developed based on imaging from patients with advanced melanoma treated with ipilimumab [23], and later applied to pembrolizumab in the same setting [26]. These irRC, based on the World Health Organization method of bidimensional measurement [33], introduced two important concepts, that of “total tumor burden” and that of confirmation of PD. Since 2009, three additional response criteria have been published by different groups, and their main features are shown alongside those of irRC in Table 1 [27,34,35]. The so-called immune-related RECIST combined some features of irRC (total tumor burden and the need to confirm PD) and of RECIST 1.1 (the use of unidimensional measurements) [34]. Later on, the RECIST group developed immune RECIST (iRECIST), which differs from previous guidelines in that (1) unconfirmed PD (iUPD) leads to “resetting of the bar” for the assessment of progression; and (2) rather than being incorporated into the total tumor burden, new lesions comprise a new set of lesions assessed in parallel to the original ones [35]. Finally, immune-modified RECIST (imRECIST) has been developed using imaging studies from patients with non-small-cell lung cancer (NSCLC) and urothelial carcinoma treated with atezolizumab (Table 1) [27].

#### 2.2.3. Duration of Response as an Endpoint

Although objective responses may be the first step towards obtaining favorable long-term results from immunotherapy, and in some cases the means to achieve symptomatic improvement, ultimately there is an expectation that such responses will be durable and improve PFS, OS, and quality of life. There is empirical corroboration for that expectation in oncology at least from the fact that several anticancer agents that received accelerated approval based on tumor responses eventually had their long-term benefit confirmed later on [36]. Some immunotherapy agents have received first approval based on responses observed in early-phase trials, and regulators have expressed interest in increasing our understanding of the dynamics and role of response-based metrics and their association with OS and quality of life [14]. The oncolytic viral therapy talimogene laherparepvec was approved on the basis of a phase 3 trial that demonstrated improvements in durable response rate, a binary endpoint defined as the percentage of patients with CR or PR lasting at least 6 months [37]. 

Early in their development, it became apparent that cancer vaccines and CPIs were associated with responses that would last several weeks or months in cancer types and lines for which this had been rare with chemotherapy [38]. Likewise, chimeric antigen receptor (CAR) T cells may lead to prolonged responses in hematological malignancies [39]. In fact, prolonged responses appear to be more specific to immunotherapy than to other treatment types. Moreover, prolonged periods of SD can also be seen as an important benefit of immunotherapy [1,40]. Finally, long-term survivors may have had SD or even PD as their best responses to immunotherapy [41,42], and in some patients, especially those with melanoma, responses have improved over time even without subsequent treatment [42,43]. Given the above considerations, duration of response (DOR) is an important element in assessing treatment benefits with immunotherapy, and several trial reports have highlighted this fact [43,44,45]. Of note, a statement by the American Society of Clinical Oncology and the Society for Immunotherapy of Cancer recommends that swimmer plots be used to depict the treatment course of individual patients, including for DOR [46]. 

### 2.3. Unique Patterns of Survival Distribution

Another early observation in the development of CPIs, in this case during comparative trials, was the unusual behavior of Kaplan–Meier curves, especially regarding a late separation and an apparent plateau in the tail of the curves. A third unusual phenomenon became apparent later, albeit less frequently: the crossing of survival curves [47]. The mechanism of action of immunotherapy was summoned as one of the potential explanations for delayed treatment effects, a phenomenon that is frequent [48,49,50,51,52,53] but not universal [54,55]. In some cases, an early detriment from immunotherapy, manifested as crossing of the curves a few months after randomization, may result from delayed effects, but hyperprogression is also a potential explanation [12]. Likewise, crossing curves may reflect the existence of subpopulations with differential effects from treatment, as seen with some targeted agents [56]. Finally, OS curves that tend to a plateau can lend support to the hypothesis that some patients are being cured [5], but may also represent the natural history in patients with indolent disease [42]. 

The above observations suggest that designing and analyzing clinical trials with immunotherapy using conventional models that assume exponential distributions and proportional hazards (i.e., a constant hazard ratio [HR]) is problematic, especially when comparisons are made with treatments from other classes [10,25]. The problems of using such methods in settings characterized by non-proportional hazards are the potential loss of statistical power [5], wrong conclusions from interim analyses [57,58], and difficulties in understanding and communicating treatment benefits [59,60,61,62]. Of note, non-proportional hazards have been found in nearly 50% of phase 3 trials of immunotherapy [63]. 

### 2.4. Prognostic, Predictive, and Response Biomarkers

Biomarkers can be of many types [64]. A biomarker associated with the likelihood of a clinical event, such as disease progression or death, regardless of treatment type, is a prognostic biomarker. A biomarker that identifies patients more likely to benefit from a specific treatment is a predictive biomarker. This distinction is important because precision medicine relies on predictive biomarkers for treatment selection. As a general rule, predictivity implies treatment-by-biomarker interaction, which indicates that the magnitude of the treatment effect varies significantly—quantitatively or qualitatively—depending on the level of the biomarker [65]. Unlike targeted therapy, for which truly predictive biomarkers have been of paramount importance in defining indications [56], the predictive role of biomarkers such as programmed cell death 1 (PD-1) and its ligand, PD-L1, remains unclear. This can be illustrated by studies in advanced NSCLC. Although PD-L1 is prognostic in this setting—since patients with PD-L1-positive disease treated with chemotherapy have better OS than their PD-L1-low or -negative counterparts undergoing the same treatment [66]—predictivity has not been convincingly demonstrated for this biomarker. This is mainly because phase 3 trials have seldom provided the evidence required for predictivity, namely that the HRs differ significantly across levels of the biomarker. In some pivotal trials, enrollment was restricted to patients with positive expression, thus precluding full assessment of predictivity [54,67]. When PD-L1-negative patients were allowed, usually because the biomarker was assessed retrospectively, predictivity was usually not demonstrated [52,68,69]. In the few cases for which an interaction was found, the level of expression defining predictivity was unclear (e.g., <1% vs. <5% expression [47]). Given these findings, subsequent trials have often enrolled only patients with PD-L1-positive disease, thus precluding proper assessment of predictivity [70,71]. As a result, current labels for CPIs display a variety of indications for which the role of PD-L1 expression is essentially dictated by the design of the corresponding trials, not by the biology of the biomarker. At present, the clinical utility of PD-L1 testing largely depends on cancer types and treatment settings [72]. The uncertainty about PD-L1 is increased by the existence of different assays and the fact that different CPIs have indications associated with different companion and complementary diagnostics. Results of these tests are influenced by (pre-) analytical factors, scoring algorithms, between-laboratory variability, and tissue type/quality, which means that eligibility for CPI treatment for the same patient can be different based on different tissue samples or the same sample being tested on different days or at different laboratories. Besides the approved companion diagnostic assays that are all based on immunohistochemistry (IHC), other assay types are available that are promising in terms of predicting clinical response to anti-PD-1/PD-L1 therapy. More specifically, multiplex ICH/immunofluorescence assays were shown to exhibit a significantly higher diagnostic accuracy than PD-L1 IHC or gene expression profiling for predicting clinical response to anti-PD-1/PD-L1 therapy. Even this best-in-class assay type still performs sub-optimally, with average sensitivities and specificities of 60% (95% CI, 53–66%) and 78% (95% CI, 73–82%), respectively [73]. Moreover, PD-L1 expression is inducible and may vary over time according to changes in the tumor microenvironment [11]. Finally, the uncertainty is made worse by the fact that CPIs were used as single agents in some NSCLC trials and combined with chemotherapy or another CPI in others, and predictivity may depend on the type of regimen. There seems to be evidence for tumor mutational burden (TMB) and tumor-infiltrating lymphocytes (TILs) as prognostic or predictive biomarkers; discussion of the corresponding literature is beyond our scope here, but we refer the reader to selected work on these topics [74,75,76,77]. Of note, microsatellite instability and mismatch-repair deficiency are associated with responses to and benefit from CPI therapy and should be taken into account in trial design [78,79,80]. In summary, trial design with CPIs may benefit from patient selection or stratification based on PD-L1 expression, TMB, TILs, or other prognostic or predictive biomarkers, but decisions need to be made individually according to the specific setting. 

Response biomarkers are used to show that a biological response has occurred in a treated patient, a concept closely linked to pharmacodynamics [64]. Immunotherapy can rely on a variety of response biomarkers that indicate an immune effect, usually mediated by activation of T lymphocytes or other effector-cell populations. Some of these potential response biomarkers are tissue-based, and others can be assessed in circulation. A discussion of these biomarkers is beyond our scope, but correlative studies are often included in trials of immunotherapy and should be considered when feasible, since pharmacodynamic studies may help to define optimal doses given the frequent absence of dose-limiting toxicity (DLT) in early-phase trials [7,81]. As pointed out elsewhere, however, pharmacodynamics is rarely a base for clear-cut decisions in the development of immunotherapy [11]. 

### 2.5. Surrogacy Issues

Statistical validation of surrogate endpoints that can reliably replace a final endpoint of interest (such as OS) is best made by assessing whether the potential surrogate is strongly associated with the final endpoint at the patient level and whether the treatment effect on the surrogate reliably predicts the treatment effect on the final endpoint at the trial level. The latter condition requires strong associations between treatment effects (e.g., between the HRs for PFS and OS). These two levels of assessment are called “patient-level surrogacy” and “trial-level surrogacy”; the former is an assessment of the surrogate as a prognostic factor, and the latter is the main prerequisite for replacing the final by the surrogate endpoint [82]. Of note, strong associations are denoted by values of the coefficient of determination (R^2^) close to 1.00.

With chemotherapy and targeted therapy, there is often a strong patient-level and trial-level association between ORR and PFS [83,84]. On the other hand, the trial-level association between ORR and OS [83,84,85] and between PFS and OS is usually weak or moderate [83,86,87,88,89]. Several authors have attempted to quantify the association between ORR and long-term endpoints and between PFS and OS in trials of CPIs [20,90,91,92,93,94,95]. Unfortunately, none of these studies used individual-patient data (IPD) meta-analysis, the most reliable approach to surrogate validation. Weak or moderate associations were generally found between ORR and both PFS and OS, as well as between PFS and OS, but interpretation of these results is made difficult by the heterogeneity of trial designs and comparators. A moderate association was found between the treatment effects on ORR and on PFS in various cancer types treated with different CPI-based regimens (R^2^ = 0.47; 95% confidence interval [CI], 0.03–0.77) [91], whereas in NSCLC a stronger association was reported in the comparison between CPI-based regimens and chemotherapy (R^2^ = 0.84; 95% CI, 0.72–0.95) [93]. In an IPD analysis conducted by the FDA on 13 trials of immunotherapy in various indications, trial-level associations were weak between ORR and OS (R^2^ = 0.13; 95% CI not given) and between PFS and OS (R^2^ = 0.13; 95% CI not given) [96]. The trial-level association between PFS (assessed using RECIST 1.1 and iRECIST) and OS was also analyzed by the FDA in their review of approved agents mentioned earlier [21]. For both criteria, the association was weak (respectively R^2^ = 0.28; 95% CI not given and R^2^ = 0.26; 95% CI not given). Thus, more work is needed to define reliable surrogates for OS with CPIs.

## 3. Some Key Decisions in Trial Design

### 3.1. Early Phase Trials

#### 3.1.1. Single-Arm vs. Randomized Trials

Phase 1 trials, phase 1/2 trials, and phase 1 trials with expansion cohorts are becoming somewhat indistinguishable in this era of precision medicine and immunotherapy. This may be a good evolution, as long as patient safety is ensured, with the goal of expediting drug development [97]. These are usually single-arm trials, following a long tradition in oncology. Likewise, phase 2 trials often have a single arm, but randomized phase 2 trials have increased in frequency over the last two decades. Given that expansion cohorts have the same main objective of phase 2 trials, namely the preliminary assessment of activity, the following discussion applies mostly to these two designs.

Single-arm trials are prone to selection bias, given that their usually strict selection criteria often lead to better results than in subsequent phase 3 trials of the same treatment [98]. Moreover, the historical data needed as assumptions for sample-size calculation are often unreliable or unavailable in the specific subset of interest in an expansion cohort, especially in the era of biomarkers. Therefore, a randomized comparison with a control treatment considered as standard of care can be a valuable tool in deciding whether the results from an early-phase trial warrant continued development of a new treatment. Moreover, randomization is a prerequisite for the assessment of predictivity, as discussed above [65]. Although early-phase trials may provide insufficient power for tests of interaction, they may provide early hints that will help develop both the drug and the biomarker. We therefore favor randomized trials in the early phases of drug development.

An ideal situation in this setting would be a randomized trial with a formal comparative intent. This could be between two or more versions of the experimental agent (e.g., different doses, schedules, or combinations) or, preferably, against true standard of care [99]. Nevertheless, financial constraints might prevent the implementation of this ideal design, even with the use of less stringent type-I and type-II error rates. An intermediate solution would be a randomized trial with no formal comparative intent. This can be performed in different ways, but a useful procedure is to compute the sample size based on the requirements for the experimental arm and to randomize a number of patients to the control arm using an equal (1:1) or unequal (e.g., 2:1) randomization. The latter reduces the number of patients treated in the control arm, for which more information is already available. Therefore, the number of patients in the control arm is not dictated by statistical requirements, as there is no formal comparison between arms at the end of the study. Rather, the control arm serves as an internal calibrator that is superior to historical controls. In a single-arm trial, an expected ORR is assumed for the experimental arm based on the historical ORR for the control arm. At the end of the trial, three possible results can ensue: a promising, a disappointing, and an outstanding ORR, always in reference to the historical ORR. These conclusions are at the risk of selection bias and might change if an internal calibrator arm is available. Depending on the performance of the control arm (as expected, better than expected, or worse than expected), the interpretation of the results for the experimental arm may change, and this may provide valuable information about the continuation of the development program.

#### 3.1.2. Defining Eligibility

The rapid pace of drug development makes the definition of eligibility criteria for immunotherapy trials a moving target, with ever-new indications and changing standards of care. Moreover, the sheer number of ongoing trials of immunotherapy makes patient enrollment increasingly challenging. Nevertheless, decisions must be made with the available information at the time of trial design and sometimes via protocol amendments. In addition to cancer type and line of therapy, as well as intrinsic patient features (such as performance status), the expression level of prognostic or predictive biomarkers may need to be considered. As discussed earlier, there is considerable uncertainty about predictive biomarkers for immunotherapy, but a presumption of this predictivity underlies the design of basket and umbrella trials [100]. Basket trials are an option for immunotherapy trials when a biomarker also represents a therapeutic target, in which case distinct disease entities (e.g., different histological types) can be assessed in expansion cohorts. Interestingly, the concept of a basket trial has been informally extended in the case of immunotherapy because the expression of the biomarker (a characteristic feature of basket trials for targeted therapy) is not always required [101,102]. When different patient cohorts are assessed in early-phase trials, the key decision, both for sample-size calculation and for interpretation of results, is whether one wishes to obtain overall or cohort-specific efficacy measures, since different statistical methods need to be used accordingly.

#### 3.1.3. Dose-Escalation Schemes

An important decision for early-phase trials of chemotherapy and targeted therapy is the choice of a dose-escalation scheme, which is usually the choice among rule-based, model-assisted, and model-based designs. An in-depth discussion of dose-escalation schemes is beyond the scope of this review but can be found elsewhere [103,104]. All these designs are premised on the need to find DLTs and determine the maximum tolerated dose (MTD). However, early-phase immunotherapy trials focusing on tolerability and safety, particularly when assessing CPIs, have been characterized by frequent absence of DLTs and predominance of toxicity after the usual DLT-assessment period of 1 month; as a result, in several phase 1 trials of CPIs the size of dose cohorts were determined based on various considerations and with sample sizes typically ranging between less than 10 and nearly 300 patients [7]. In consequence, the maximum administered dose rather than the MTD has typically been used to guide the selection of the recommended dose for subsequent development. Thus, the choice among the available designs should be based on several considerations, among which the planned number of cohorts, the expected therapeutic window, the solidity of preclinical or previous clinical evidence regarding the dose–response and dose–toxicity relationships, and the knowledge about agents to be combined with the experimental drug when that is the case. From a statistical standpoint, key considerations are the operating characteristics of each design in terms of expected and maximum sample size, the probability of overdosing patients, and the probability of correctly selecting the recommended phase 2 dose. Moreover, it is important to consider the need for dose optimization in early-phase trials, following recent FDA views on this topic; dose optimization in this sense refers to a joint assessment of safety and efficacy [105,106]. This may entail the identification of the optimal dose(s) prior to/concurrently with establishing safety and efficacy and before the start of a registration trial, as well as adequate characterization of the pharmacokinetic properties of the agent(s).

A topic intimately related to the choice of design for phase 1 is the methodology of safety assessment, discussed below.

#### 3.1.4. Safety Assessment

The unique safety profile of CPIs, with the predominance of IRAEs and few of the typical adverse events seen with other modalities, has led to several initiatives and the upcoming update of the Common Terminology Criteria for Adverse Events (version 6.0) [19,107]. The key decision regarding safety when designing phase 1 immunotherapy trials is the definition of the DLT assessment period. Although most IRAEs have their onset within the first 3 months of treatment, very few can be detected during the conventional DLT period comprising the first cycle. Therefore, consideration should be given to extending that period, in order to incorporate late-onset adverse events and DLTs in the definition of the recommended phase 2 dose. If this is deemed not feasible, all observed IRAEs at the end of the dose escalation can be taken into account when modeling the dose-toxicity relationship and selection of the MTD. For phase 1 and subsequent trials, the protocol should be clear on the definitions and management provisions for IRAEs. Moreover, special considerations are required for trials of CAR T cells and other immunotherapy modalities marked by unique toxicity, including cytokine-release syndrome and neurological symptoms [108,109].

#### 3.1.5. Efficacy Assessment

Neither PFS nor OS are very useful as primary endpoints in early-phase trials, given the typical absence of a control arm and the lack of statistical power in small studies. Thus, ORR and DOR are typically the primary mode of assessing efficacy in these trials. The definitive role of immunotherapy-related response criteria, which have not been validated on a large scale, has not yet been defined. The application of these criteria is more time-consuming, and one should have in mind that they increase the final ORR by only 1% to 2% in many cases, with an additional 5–10% of patients who would have PD by RECIST 1.1moving to the SD category [21,23,27,110,111,112,113]. On the other hand, patients may move from RECIST 1.1-defined PD to SD, PR or CR when treated beyond progression, something that seems more likely in advanced melanoma and renal-cell carcinoma [114,115,116].

The RECIST group recommends that phase 3 clinical trials should continue to use both RECIST 1.1 and iRECIST, with the former used to define primary response-based outcomes, and iRECIST used as the primary criteria in early-phase trials [35]. The decision to use RECIST 1.1 or iRECIST depends mainly on practical considerations regarding the added complexity of implementing the latter and on whether pseudoprogression is relevant in the specific disease setting. A sensible provision in clinical trials for which ORR is the primary or an important secondary endpoint is that responses be assessed using RECIST 1.1 as the primary response criteria and that physicians be given the liberty to pursue treatment beyond RECIST 1.1-defined PD. If iRECIST is used as primary criteria, the potential problem is the frequency of inconclusive cases (i.e., iUPD) if many physicians forgo treatment beyond progression. With FDA-approved agents, two-thirds of patients with RECIST 1.1-defined PD had iUPD, and only one-third had confirmation of PD by iRECIST [21]. If one wishes to quantify the frequency of pseudoprogression, iRECIST can be used as secondary response criteria, with patients treated beyond progression contributing to such quantification. For selected disease settings characterized by higher rates of pseudoprogression, iRECIST should be the primary criteria, particularly with novel agents in early-phase trials.

Although the assessment of DOR is straightforward when made descriptively, the comparison of DOR between treatments is problematic. This comparison is potentially biased because it only considers responding patients; as a result, the comparison is made based on a post-randomization feature, thus violating the intention-to-treat principle. As a corollary to this problem, DOR cannot be used as the basis on which the sample size is justified for a clinical trial, whether or not it is randomized. Interestingly, the treatment that leads to more frequent responses will usually have responding patients with worse prognosis, and in fact the bias may be against the superior treatment [117]. Therefore, DOR reported in the literature should be seen as exploratory, particularly when groups are compared. Nevertheless, the problem of bias can be mitigated to some extent when such an analysis is conducted with statistical techniques that control the analysis-by-responder bias, something arguably more relevant for late-phase trials [118,119]. The first of these procedures consists of generating more comparable groups by either (1) removing responding patients with the least tumor shrinkage from the group with more responders or (2) adding non-responding patients with the most tumor shrinkage to the group with fewer responders, in both cases maintaining similar proportions of responders in both groups [118]. Another proposed method is one that takes advantage of the additive properties of restricted mean survival time (RMST). It consists of ascribing a DOR to each patient in a trial, thus avoiding the exclusion of non-responding patients from analysis. This is possible if one constructs Kaplan–Meier curves (for each arm separately) for an artificial composite endpoint defined as the time between treatment initiation and the first among response, progression, or death. The RMST for this composite endpoint is computed for each arm and subtracted from the RMST for the corresponding PFS curve, constructed in the usual manner, thus yielding the restricted mean DOR for each treatment arm. If this procedure is used, non-responding patients will have a DOR of zero, because the same first event (progression or death) will be used to indicate the occurrence of the composite endpoint and of PFS in these patients.

### 3.2. Late-Phase Trials

#### 3.2.1. Conventional vs. Adaptive Trials

In most cases, conventional (i.e., without adaptations) trial designs are preferable in the attempt to formally compare two treatments, given their relative simplicity, well-known statistical characteristics, logistical ease of implementation, and more straightforward interpretation. In some cases, however, adaptive trials may be warranted. These are defined as clinical trials that allow for prospectively planned modifications of one or more aspects of their design based on accumulating data from the trial [120]. Therefore, unplanned changes based on interim results, as well as protocol amendments, are not considered adaptations. A discussion of the various types of adaptive trials and design elements amenable to adaptation is beyond our scope here, but the reader should note that excellent reviews are available [121,122] and that consultation with authorities is advised when planning an adaptive trial with a potential role in regulatory submission [120]. When multiple experimental regimens can be compared with a common control arm, the specific type of adaptive trial known as multi-arm, multi-stage design should be considered; this and other “platform” designs can bring far greater efficiencies than simpler adaptive trials [100]. Given the large number of agents being developed in combination or comparison with CPIs, these efficiencies should be kept in mind in the attempt to allocate patients and resources rationally.

#### 3.2.2. Choice of Primary Endpoint

In the chemotherapy era, and to a great extent with targeted therapy, a long debate has prevailed between the merits of PFS and those of OS when choosing the primary endpoint in phase 3 trials for advanced disease. Given the limitations of OS, PFS eventually became the most frequently used primary endpoint, particularly in the first line [123]. With direct cytotoxic modalities, the effects of treatment occur during its administration; conversely, immunotherapy displays putative delayed effects, which combine with the unusual patterns of response to raise doubts about the worth of PFS in this setting. Indeed, in many cases a discrepancy was noted between treatment effects on PFS and OS, with gains in the latter unaccompanied by gains in the former [47,51,52,53,55,67,124]; likewise, a meta-analysis of 94 randomized trials used by the FDA for drug approvals between 2011 and 2017 (13 on immunotherapy and 81 on other modalities) showed that PFS benefit with immunotherapy (quantified by RMSTs) was less than with other modalities, and vice versa for OS benefits [125]. An initial increase in tumor volume from immune infiltration, delayed anticancer activity, or a sustained effect beyond progression have been proposed as explanations for that discordance [52]. Both PFS and OS play key roles in late-phase trials of immunotherapy; although OS appears to have been used more frequently, PFS is likely to regain relevance, particularly in comparisons between different immunotherapy regimens [126,127]. Moreover, nearly half of phase 3 trials of immunotherapy have used two primary endpoints, usually PFS and OS [128]. The key decision in these cases is whether adjustment for multiplicity is warranted; this is only the case when trial positivity is declared if results are positive for at least one primary endpoint and not when both are considered co-primary endpoints so that both have to be significant for the trial to be successful [129]. Although we do not discuss secondary endpoints here, it should be noted that adjustment for multiplicity may also be required depending on formal testing strategies proposed for secondary endpoints.

There is some evidence that using RECIST 1.1 leads to a slight underestimation of PFS, in comparison with iRECIST; in the assessment based on FDA-approved agents, the difference in medians was only 0.2 months in patients overall, but larger among those with RECIST 1.1-defined PD having treatment past progression [21]. A slightly longer, but also not clinically relevant difference of 0.5 months was found between the two criteria, in this case for average RMST for PFS, in a smaller study based on published data mostly from observational studies [112]. The relevance of these findings is still unclear, particularly in randomized trials when treatment arms are assessed in the same fashion. Likewise, the use of iRECIST may lead to longer DOR than that of RECIST 1.1 [21], since DOR may be quite long among patients receiving treatment beyond progression who eventually respond [111]. Once again, the relevance of these findings is debatable, particularly in light of the limitations of DOR for formal comparisons, highlighted above.

#### 3.2.3. Assessment of the Treatment Effect

HR is the most frequently reported relative measure of treatment effect on OS and other time-to-event endpoints. Unfortunately, HR suffers from limitations in all these respects, notwithstanding its usefulness and tradition. The statistical limitation of HR stems from the fact that its expression by a single numerical value implies that it is constant in time, thus indicating a constant reduction or increase in the hazard of the event(s) of interest. This assumption of proportional hazards is often violated in the case of immunotherapy [63]. Several methods have been proposed to deal with the limitations of HR and deviations from non-proportional hazards in the analysis of time-to-event endpoints such as PFS and OS (Table 2). Although their uptake appears to have been low, these methods should at least be considered in the design of late-phase immunotherapy trials [6,9,10,58,60,130,131,132].

Because survival time generally has a skewed distribution, and given the ease of reading the median directly from survival curves, the mean survival time has long been neglected as a measure of central tendency in survival analysis. If a survival curve reaches zero (when the longest observed time in that group is an event), the mean survival time for that group can be estimated by computing the area under its survival curve. However, this is very rare in practice. Nevertheless, it is possible to estimate the RMST by restricting (i.e., truncating) the follow-up to a given time t and analyzing the data only up to time t [133]. The RMST for a group of patients is the area under the survival curve through time t, thus measuring the average time survived by patients over the period of interest. The RMST for two groups can be contrasted by subtraction (an absolute measure) or by their ratio (a relative measure). Importantly, the use and interpretation of RMSTs does not depend on the presence of proportional hazards [133]. The RMST can be used even in the extreme cases of non-proportional hazards, when the survival curves initially overlap or when they cross, as can be observed in immunotherapy trials [60]. For these reasons, this practice has already been implemented for secondary analysis of immunotherapy trials in which hazards are non-proportional [79,134]. Moreover, RMSTs can be used to estimate treatment-free survival, a metric that can be of relevance in the exploratory analysis of immunotherapy trials [135]. Finally, immunotherapy trials can be designed with the goal of comparing RMSTs as the primary analysis, and software is available for sample-size calculation [60].

Since the assumption of proportional hazards is too strong for many situations found in practice, especially phase 3 trials of immunotherapy [136], weighted logrank tests have received renewed attention with the advent of immunotherapy [137]. Weighted logrank tests are attractive because they may give more weight to later time points, something desirable when there are delayed treatment effects [138,139,140]. On the other hand, weighted logrank tests can raise ethical concerns and provide biased estimates according to the selection of weights. When weighted logrank tests are used, the interpretation of treatment effects is still made using HRs, with the limitations already highlighted (Table 2).

Because the interpretation of treatment effects using HR is made on the hazard scale, it is impossible to translate the information about the mortality hazard reduction into a difference in survival time. Conversely, the time scale is more natural and can be explored using the accelerated failure time models. By assuming that the effect of treatment manifests itself in shortening or extending survival time, these models lead to a simple and natural interpretation of the treatment effect, which can be quantified in terms of the ratio of the mean survival time for two competing treatments. These models do not require the assumption of proportional hazards, thus being advantageous for immunotherapy trials. However, it is worth noting that the accelerated failure time model, similar to the proportional-hazards model, is not valid in the situation of a delayed treatment effect (Table 2).

From the point of view of clinicians and patients, HR is limited by being a relative measure, as it is generally accepted that absolute measures can best inform individual decisions by conveying results in a manner that improves clinician and patient understanding of trial results [62,141]. Measures used to assess the benefit of any therapy should be well-founded from the statistical, clinical, and patient perspectives [62]. The Net Treatment Benefit (NTB) is an absolute measure of treatment effect based on the technique of generalized pairwise comparisons (GPC), which compares the outcomes for every patient in the experimental arm with that of every patient from the control arm [142]. If these pairs can be classified as a “win” (the patient in the experimental group has a better outcome than the patient in the control group), a “loss” (the opposite situation), a “tie” (if there is no difference in outcome between the two individuals), or as “non-informative” (when there is censoring or missing data), the NTB is the difference between the probability of a win and the probability of a loss [142]. Importantly, the NTB allows comparisons that involve more than one outcome, as long as they can be prioritized in terms of their desirability [143]. This feature gives the NTB a patient-centricity that is of unique value. Likewise, it allows the construction of composite endpoints in which the time to the worst outcome (e.g., death) can be assessed with higher priority than the least serious one (e.g., progression) in the same endpoint. This is in contrast to PFS, which considers the outcome first occurring between progression and death. Moreover, the NTB can be used to address situations of non-proportional hazards, particularly when there is late separation of OS curves, such as in trials of CPIs (Table 2) [59,144]. The NTB is computed using IPD from randomized trials, and simulations allow the use of this methodology to compute sample sizes for trial design. Metrics related to the NTB are the win ratio—the ratio between the probability of a win and the probability of a loss [145]—and the success odds, which handles ties by assigning 50% of them to both the numerator and the denominator of the win ratio [146]. These novel metrics based on GPC are gaining increasing attention because they allow one to combine as many endpoints of any type in the analysis, which is a paradigmatic shift from conventional analyses of a single outcome at a time.

## 4. Conclusions

We have provided an overview of methodological issues related to trial design in immunotherapy, in a manner that we believe can help those working in the field. For specific issues requiring a more in-depth evaluation, the interested reader will need to consult other sources and gather additional information. Our aim has been to highlight the key considerations in trial design and for which conventional knowledge coming from other treatment modalities in oncology may not suffice. Immunotherapy has revolutionized the systemic treatment of patients with cancer, but novel methodological approaches to trial design, analysis, and interpretation are required to accommodate these issues and others that are likely to be identified in the future. As the role of immunotherapy expands, agents from this class are likely to be combined with other systemic and local treatment modalities, thus leading to a continual reappraisal of the adequacy of existing methodology.

## Figures and Tables

**Table 1:** Comparison of immune-related response criteria.

Feature	Immune-Related Response Criteria [23]	Immune-Related Response Evaluation Criteria in Solid Tumors [34]	Immune Response Evaluation Criteria in Solid Tumors [35]	Immune-Modified Response Evaluation Criteria in Solid Tumors [27]
Year of publication	2009	2013	2017	2018
Measurement	Bidimensional	Unidimensional	Unidimensional	Unidimensional
Characterization of the tumor burden	Measurements of up to 15 index lesions (up to 5/organ, up to 10 visceral and 5 cutaneous lesions) added to measurements of new, measurable lesions (≥5 × 5 mm; up to 5/organ, up to 10 visceral and 5 cutaneous lesions) to provide the total tumor burden	Measurements of target lesions (presumably following RECIST stipulations) added to measurements of new lesions to provide the sum of measurements	In addition to usual RECIST stipulations, new lesions are characterized as “new lesion target” and “new lesion non-target” (and not incorporated in the total tumor burden)	Measurements of target lesions (following RECIST stipulations) are added to measurements of new lesions (up to 5 in total and 2/organ) to provide total tumor burden; when not measurable, new lesions are not factored into the assessment of PD, unless they become measurable and the maximum of 5 measurable new lesions has not been reached
Definition of PD	Increase ≥ 25% in tumor burden compared with nadir (at any time point) in two consecutive observations at least 4 weeks apart; progression of non-index lesions does not define PD	Increase ≥ 20% in the sum of measurements compared with a nadir in two consecutive observations at least 4 weeks apart	PD can be assigned multiple times, as long as it is not confirmed 4–8 weeks later; if PD is not confirmed (i.e., tumor shrinkage is observed in comparison with baseline), the bar is reset so that it needs to occur again (compared with nadir) and then be confirmed	Increase ≥ 20% in total tumor burden compared with nadir in two consecutive observations at least 4 weeks apart; progression of non-target lesions does not define PD

PD, progressive disease; RECIST, Response Evaluation Criteria in Solid Tumors. Duration of response as an endpoint.

**Table 2:** Selected statistical methods that may improve design, analysis, and interpretation of immunotherapy trials.

Method	Advantages	Disadvantages
Restricted mean survival time	Additive properties; applicable even in the extreme cases of non-proportional hazards with initially overlapping or crossing survival functions; useful even when median survival is not reached	Dependence on truncation time; non-intuitive interpretation
Weighted logrank test	Higher statistical power in the same nonparametric framework as the logrank test	Potential bias from weight selection; ethical concern from a differential weighing of earlier and later events; loss of power if the shape of curves is incorrectly specified
Accelerated failure-time models	Interpretation in terms of the mean survival time (preferable to median survival time); robustness to omission of covariates; no parametric distributional assumptions in the case of semiparametric models	Unsuitable for the extreme cases of non-proportional hazards with initially overlapping or crossing survival functions
Net Treatment Benefit	Intuitively conveys probabilities on an absolute scale; allows different stakeholders to prioritize outcomes and thresholds of benefit; allows simultaneous assessment of several endpoints, including safety	Recently proposed, with uncertain acceptability by regulatory agencies; potential for bias when average follow-up is much shorter than the longest event time; properties such as the impact of censoring still under study; choice of priorities and clinical thresholds arbitrary
